# Trust Toward Robots and Artificial Intelligence: An Experimental Approach to Human–Technology Interactions Online

**DOI:** 10.3389/fpsyg.2020.568256

**Published:** 2020-12-03

**Authors:** Atte Oksanen, Nina Savela, Rita Latikka, Aki Koivula

**Affiliations:** ^1^Faculty of Social Sciences, Tampere University, Tampere, Finland; ^2^Faculty of Social Sciences, University of Turku, Turku, Finland

**Keywords:** trust, human–technology interaction, robot, artificial intelligence, individual differences, trust game

## Abstract

Robotization and artificial intelligence (AI) are expected to change societies profoundly. Trust is an important factor of human–technology interactions, as robots and AI increasingly contribute to tasks previously handled by humans. Currently, there is a need for studies investigating trust toward AI and robots, especially in first-encounter meetings. This article reports findings from a study investigating trust toward robots and AI in an online trust game experiment. The trust game manipulated the hypothetical opponents that were described as either AI or robots. These were compared with control group opponents using only a human name or a nickname. Participants (*N* = 1077) lived in the United States. Describing opponents with robots or AI did not impact participants’ trust toward them. The robot called jdrx894 was the most trusted opponent. Opponents named “jdrx894” were trusted more than opponents called “Michael.” Further analysis showed that having a degree in technology or engineering, exposure to robots online and robot use self-efficacy predicted higher trust toward robots and AI. Out of Big Five personality characteristics, openness to experience predicted higher trust, and conscientiousness predicted lower trust. Results suggest trust on robots and AI is contextual and it is also dependent on individual differences and knowledge on technology.

## Introduction

Robotization and artificial intelligence (AI) are expected to change societies profoundly (Borenstein, 2011; Liu and Zawieska, 2017; Makridakis, 2017). Robots and AI are expected to become more humanlike and to handle tasks normally performed by individuals (Goetz et al., 2003; Frey and Osborne, 2017). Huang et al. (2019) argued that there is an on-going shift from the current thinking economy to a feeling economy. In the longer run, AI is likely to contribute to communicating, interacting, and empathizing tasks formerly performed by humans (Huang and Rust, 2018; Huang et al., 2019). Intelligent chatbots are already a working example of this process.

We argue that analyzing trust in human–technology relationships is important to understand the transformative change brought on by AI and new-generation social robots. Trust is essential in human interactions and human well-being, and without trust, human societies would not function in a civilized manner (Putnam, 2000; Cook, 2001; Uslaner, 2002; Hardin, 2005). Trust is equally important in technological encounters (Hancock et al., 2011; Sanders et al., 2011; Schaefer et al., 2016). Existing literature shows that humans are more willing to accept new technologies, such as robots, when they have prior experience (Venkatesh, 2000; Nomura et al., 2006; Bartneck et al., 2007; Heerink et al., 2010) and self-efficacy in handling them (Hsu and Chiu, 2004; Hasan, 2006; Rahman et al., 2016; Latikka et al., 2019). There is a current need for studies investigating trust toward AI and robots, especially in first-encounter situations involving little information about the other actor.

This article reports the results based on a trust game experiment involving robots and AI. Our aim was first to analyze whether participants show lower trust toward robots and AI than toward others given only name or nickname but not described as a robot or AI. Furthermore, our study aimed to explore how trust functions in human–technology interaction, while considering different social psychological factors, such as robot use self-efficacy and personality.

### Trust in Human–Technology Interactions

Trust has psychological and sociological dimensions. From developmental psychology, researchers know that humans build basic trust toward those closest to them, and later, they are taught to understand how much and in whom they should trust (Simpson, 2007b; Van Lange, 2015). In sociology, trust is considered to be the social glue that brings people and good things in societies together (Putnam, 2000; Uslaner, 2000; Rothstein and Uslaner, 2005; Bjørnskov, 2012).

Trust can be divided into trust in people close to individuals (family, friends, and colleagues) and trust in people who are more distant from individuals (people in general or strangers). Distinctions, such as intimate versus abstract trust (Freitag and Traunmüller, 2009), thick versus thin trust (Putnam, 2000), and particularized versus generalized trust (Stolle, 2002; Uslaner, 2002), characterize these different dimensions of trust. Intimate trust is universal, whereas generalized trust depends on the circumstances. In their seminal work on trust in automation, Lee and See (2004) defined trust “as the attitude that an agent will help achieve an individual’s goals in a situation characterized by uncertainty and vulnerability” (p. 51).

Advanced technology, such as AI, causes complex challenges, due to their intelligence and potentially hidden motivations. Simple machines of previous decades could be trusted on the basis that they worked as expected, but intelligent machines, such as new robots or other AI solutions, cause potential concerns. On what premise should people trust them? Who programmed them? What have they learned from humans already? Are their purposes good or bad? These questions are one reason that the ethics of AI are currently being discussed intensively (Russell et al., 2015; Dignum, 2018; Winfield et al., 2019). There is, however, nothing new in these discussions, as similar concerns have been raised before. For example, computers were suspected to have negative effects on humanity and society (Simon, 1977), and indeed, they have been found to cause anxiety for some (Fariña et al., 1991).

A literature review by Sanders et al. (2011) showed that robot type, functionality, level of automation, and personality impact how they are trusted: People show higher trust when a robot looks as expected and when certain anthropomorphic features, such as gestures and emotional expressions that are likely to increase trust, are included. Also, robot personality is associated with trust, and people tend to trust robots that have likeable features. People tended to more often trust robots that showed more positive emotion (Mathur and Reichling, 2016). In a recent experiment, a robot apologizing for its mistake was considered more likeable, but less capable. Likability and warmth-based trust had a positive effect on intentions to use the robot again (Cameron et al., 2021).

Robots are typically defined by their physical characteristics (see, for example, International Organization for Standardization [ISO], 2014), but AI can function via any technological apparatus and it is more hidden and integrated. In contrast to physical robots, the concept of bot refers to online agents and software applications that can also use AI and simulate human interaction, for example intelligent chatbots (e.g., Mitsuku and Alisa). Previous studies have shown that an extensive amount of visual images of robot affects how they are perceived (Fortunati et al., 2015). This may cause trust toward AI to be more abstract than trust toward robots. Another possible influencing factor consists of the differences between how fictive robots are represented and how real robots are designed. People have become familiar with fictive robots, such as R2-D2 and C3PO in *Star Wars*, and real ones, such as Paro and Nao, who embody harmless and pet-like appearances. In contrast to these, AI is often portrayed as a higher-level operator in popular fiction, such as in 2001*: A Space Odyssey* by Stanley Kubrick and Her by Spike Jonze.

Studies on computer interfaces show that people often treat computer interfaces as though they are human, especially in the research on the computers are social actors paradigm (Reeves and Nass, 1996; Nass and Moon, 2000). Based on research conducted since the 1990s, introducing familiar characteristics to technology seems to be decisive in computer interface success, as people are more willing to be drawn toward others who are similar to them (Reeves and Nass, 1996; Nass and Moon, 2000; Nass and Lee, 2001). This similarity–attraction hypothesis has been widely tested in social psychology (Montoya and Horton, 2013).

Trust always has a social and operational context or environment. How much people trust technology, such as robots and AI, depends on where they are used. Trust as a behavior (B) is a function of a person (P) and their environment (E), including the object to be trusted, according to Levin, 1935, p. 73) classic equation of factors explaining behavior: *B* = *f*(*P*, *E*). For example, people may consider service robots in hospitals trustworthy if they trust hospitals in general. Situational and environmental aspects may also influence the trust people have. In some situations, however, it is challenging for people to judge the intentions of other people who have designed robots or AI. These types of situations may occur for people who browse online and meet chatbots or other agents using AI.

Aside from previously stated robot-related aspects, human-related factors impact the extent to which people trust robots and AI (Hancock et al., 2011; Schaefer et al., 2016). Older people trust robots and other automated processes less than younger people (Hoff and Bashir, 2015), which is consistent with the findings showing that older people harbor more negative emotions toward robots and are more reluctant to support the use of AI or service robots (Scopelliti et al., 2005; Eurobarometer, 2012; Zhang and Dafoe, 2019). Previous studies have not found consistent differences between genders regarding trust toward robots (Hoff and Bashir, 2015). However, women tend to show more negative attitudes toward robots and are less willing to work with robots (De Graaf and Allouch, 2013; Reich and Eyssel, 2013; Reich-Stiebert and Eyssel, 2015).

Employment status, household income, and educational background are factors that determine people’s access and usage of new technologies (Van Deursen and Van Dijk, 2014), and they are crucially linked to how much people trust other people (Delhey and Newton, 2003). Recent research on human–technology interactions has shown that people’s attitudes toward robots and AI vary according to employment status and household income (Gnambs and Appel, 2019; Zhang and Dafoe, 2019). Education and interest in technology are also essential factors behind acceptance of and confidence in new technologies (Heerink, 2011).

Robot use self-efficacy has a potential impact on trust. Self-efficacy refers to an individual’s beliefs about their ability to perform in a particular situation or task (Bandura, 1986, 1997) and has been studied throughout the history of technological advances (Compeau and Higgins, 1995; Agarwal et al., 2000; Hasan, 2006; Rahman et al., 2016), including the Internet (Eastin and LaRose, 2000; Hsu and Chiu, 2004). In the context of robot-based technology, robot use self-efficacy has been found to be a separate construct from general self-efficacy and has been found to predict the acceptance of robots in a health care context (Latikka et al., 2019; Turja et al., 2019).

Last, personality traits impact trust and the ways in which people use technology. Personality impacts, for example, what kinds of robots people find likeable and trustworthy (Sanders et al., 2011; Correia et al., 2019). Currently, the five-factor model of personality (the Big Five) is most widely used and accepted (Digman, 1990; John et al., 2008). Studies on trust and personality show that high agreeableness has a positive relationship with high interpersonal trust (Mayer et al., 1995; Mooradian et al., 2006). Some evidence also suggests a positive correlation between trust and openness (Kaplan et al., 2015). Personality has been noted in studies on trust and technology, but findings remain limited (Hancock et al., 2011; Schaefer et al., 2016). Evidence suggests that extroverts are more receptive of robots and that low neuroticism is connected to the acceptance of robots (Robert, 2018). Another study found a relationship between increased trust in automation and high agreeableness or conscientiousness (Chien et al., 2016).

### Measuring Trust With a Trust Game

The trust game is an experimental method of measuring trust as investment decisions. It originates from the investment game, originally introduced by Berg et al. (1995), in which trust and reciprocity are assessed in an economic exchange relation. Previous studies have indicated that people’s motivation to reciprocate trust is determined not only by maximization of personal goals but also by consideration of consequences for both self and others (Fehr and Gintis, 2007; Van Den Bos et al., 2009). In the trust game, the consequences of trust are determined concretely by the amount of money that participants are willing to give up (Berg et al., 1995; Evans and Revelle, 2008).

The literature on trust research includes many variations of the trust game (Trifletti and Capozza, 2011; Samson and Kostyszyn, 2015; Xin et al., 2016). In general, the player receives a certain amount of money and chooses how much of that money to send to the opponent. The money received by the opponent is multiplied and the opponent can decide to keep the money or return part or all of it. The amount of money transferred, if any, measures the investment decision, that is the trust behavior. In a simpler format, the player receives a certain amount of money and decides how much of that money to give to the described opponent (Berg et al., 1995; Evans and Revelle, 2008).

Results of the trust game have been shown to correlate with trust and are thus not limited solely to economic decisions (Dunning et al., 2012) or altruistic behavior (Brülhart and Usunier, 2011). However, measuring trust is broadly discussed and challenged because trust has such a wide variety of definitions (Hardin, 2005). The trust phenomenon is complex and consists of three to four parts as *A trusts that B is/makes X*, to which condition Z can be added. Changing any of these dimensions may have an effect on the resulting trust (Simpson, 2007a). Furthermore, there is also a paradox of information in trust: When trust presupposes a lack of information, it is also based on information (e.g., on experience and conditions), which may lead to difficulties in examining the degree of trust (Nooteboom, 2011).

However, trust can evolve from expected reciprocity (Ashraf et al., 2006). Reciprocity, in turn, is expected more when the cues for personal identity are present. At a group level, reciprocity is expected more from ingroup members than outgroup members when social identity is salient (Tanis and Postmes, 2005). The use of a person’s first name as a relatively minimal social cue enables the generation of positive interpersonal impressions (Tanis and Postmes, 2003).

The trust game is adaptable to different studies on societal and psychological phenomena, such as usage and trust of new technologies. The trust game was originally developed in the context of investment decisions (Berg et al., 1995), which limits the perfection of its fit to other areas. Further, as a two-player one-time game it may not capture all complex dynamics around the decisions to trust (Camerer, 2003, p. 85; Dunning et al., 2012). However, trust measured via survey items associates positively with investing money in the trust game (Evans and Revelle, 2008). The trust game is popular among trust scholars (Johnson and Mislin, 2011), and useful for experimental research designs that aim to understand contextual variations of trusting others. The benefit of the trust game, as an experiment, is its measurement of actual behavior, which may give a relatively reliable indication of how people function in a real-life context. Despite previous studies on trust in technologies (Hancock et al., 2011, 2020; Schaefer et al., 2016), many of these studies have focused on using traditional survey measures (e.g., Yagoda and Gillan, 2012) and only a few studies used experiments to investigate trust in technologies (e.g., Correia et al., 2016, 2019; Ferreira et al., 2016). The trust game has not been utilized to analyze trust in robots and AI before.

### This Study

This study tested whether participants show trust in robots and AI and it used an experimental trust game design. Our research question was: Do participants trust robots or AI less than control group members not specified as a robot or AI? The trust game manipulated the hypothetical opponents that were described as either robots or AI. These were compared with control group opponents using only human name or nickname. The main hypotheses of the study were preregistered at the Open Science Framework before collecting the data (Oksanen et al., 2019).

The hypotheses were based on a similarity–attraction hypothesis underlining that people are more likely to be drawn toward those similar to them (Montoya and Horton, 2013). Further, based on the existing literature, we expected that participant would show lower trust on robots and AI, because they are still emerging technologies and people are not necessarily familiar with their operational logic and intentions. In other words, our hypotheses were based on trust research indicating that people show more trust toward things that they are familiar with (Gefen, 2000; Hancock et al., 2011). Originally the hypothesis pre-registration specified control group opponents as humans, but we updated this to unspecified control group to reflect the fact that growing relevance of different AI agents could also lead people to interpret the control group opponent as non-human. Our hypotheses are then as follow:

H1: Respondents trust robot opponents less than control opponents who are not specified as either human or non-human.

H2: Respondents trust AI opponents less than control opponents who are not specified as either human or non-human.

H3: Respondents trust opponents with a human name more than opponents with a nickname.

The second part of the analysis focused on investigating the correlations of the trust expressed in the trust game. The aim was to analyze individual differences in trust of robots and AI. We expected that technology education (H4), robot exposure online (H5), and robot use self-efficacy (H6) would predict higher trust toward robots and AI. These were generally based on studies on trust showing that trust is grounded in personal social interaction experiences (Van Lange, 2015) and empirical evidence on trust toward technology, automation, and robots (Hancock et al., 2011; Schaefer et al., 2016). Additionally, we expected (H7) that personality traits, such as agreeableness, conscientiousness, openness, and extraversion, would have a positive relationship and that neuroticism would have a negative relationship with trust toward robots and AI (Mayer et al., 1995; Mooradian et al., 2006; Kaplan et al., 2015; Chien et al., 2016; Robert, 2018).

## Materials and Methods

### Participants

Data were collected in April 2019 from American participants (*N* = 1077, 50.60% female, *M*_*age*_ = 37.39 years, *SD*_*age*_ = 11.42 years) who were recruited via Amazon’s Mechanical Turk, which is considered a reliable source to obtain research participants in the United States (Buhrmester et al., 2011; Paolacci and Chandler, 2014; Huff and Tingley, 2015). Recent analysis also showed that the financial situation of MTurk participants mirrors that of the United States and that respondents do not find requesters abusive (Moss et al., 2020). Some concerns have arisen, however, due to non-United States residents trying to access the surveys intended for United States residents only. We used the procedure suggested by Kennedy et al. (2020) and excluded non-United States participants from the data. We also checked the data for respondents with odd response behavior (e.g., who finished the survey too quickly or selected the same answer option throughout the survey).

The participants were from 50 states, with the highest response rates coming from California (8.08%), New York (7.89%), Florida (7.80%), and Texas (7.06%). The population was 72.52% White (not Hispanic), 6.22% African American (not Hispanic), 13.09% Hispanic, and 5.94% Asian. These figures are close to the United States population estimates, except that the African American population was under-represented (U. S. Census Bureau, 2010). In addition, participants have, on average, higher educational attainment than the population in general, as 66.07% of participants 25 years and older had a college degree, but only 39.20% in the population have achieved this education level. Higher educational attainment of Mechanical Turk respondents has been previously noted (Huff and Tingley, 2015; Hitlin, 2016).

### Procedure

In this between-subjects design study, survey respondents were asked to provide sociodemographic and personality information before entering the experiment. After the experiment, they were asked about their experience in using robots and their robot attitudes. Median response time for the whole survey, including the experiment, was 8 min and 10 s (*M* = 9 min and 58 s) and 44 s for the experiment only (*M* = 1 min and 9 s). Survey respondents received a small reward of $0.90 for their participation. The academic ethics committee of Tampere Region in Finland stated, in December 2018 (statement 89/2018), that the research project did not involve ethical problems.

At the beginning of the trust game, the participants (i.e., players) were told of a hypothetical situation in which they were given $1,000 and could decide to keep the whole sum or share part of it with their opponent. They were told that the experimenter would triple the amount of money they gave (i.e., if they gave $500, the other player would receive $1,500). Then, they were told that their opponent could freely decide whether to return any money to the participant player. At the end of the explanation, we asked the participants to fill in a box containing an amount between $0 and $1,000 (see Appendix A). The opponent did not have to take action in this hypothetical situation; only the participant was asked to choose an action.

Respondents were randomly assigned to one of six groups at the beginning of the experiment. The trust game included manipulation of hypothetical opponents; they were described as either AI or robots and compared with control group opponents, which used only human names or nicknames. Opponents were introduced as robots (Michael or jdrx894), AI (Michael or jdrx894), or only by name (Michael or jdrx894). We carried out the manipulations by adding the words “robot” or “artificial intelligence”—for example, “opponent name: jdrx894, a robot” or “opponent name: jdrx894, an artificial intelligence.” The experimental conditions were compared with two control group opponents who were introduced with only a human name (Michael) or a nickname (jdrx894) without calling them robots or AI.

An analysis of the basic characteristics of the robot (Michael: *n* = 192; jdrx894: *n* = 172) and AI (Michael: *n* = 185; jdrx894: *n* = 171) experimental groups and control groups (Michael: *n* = 171; jdrx894: *n* = 186) showed that the randomization was successful and that no statistically significant differences in gender, age, education or Big Five personality traits were present.

### Measures

#### Trust

The amount of money given to the opponent in the trust game was the outcome variable, considered to measure a participant’s trust toward their opponent. This variable ranged from $0 to $1,000. The mode value for all groups was $500. There were no issues with skewness in any of the six groups (from 0.04 to 0.37), but kurtosis figures indicated light-tailed distribution (from 1.97 to 2.39 when 3 equals the normal distribution). A Shapiro–Wilk test showed that only one group out of six was normally distributed.

#### Sociodemographic Variables

We used sociodemographic variables as independent variables in the second part of the study. They included age, gender, employment status, household gross annual income, and degrees in technology or engineering.

#### Robot-Related Variables

The second part of the study included also several variables on robots. Exposure to robots online was measured with the question, “Have you heard, read, or seen anything about robots in social media, internet forums, or blogs?” (*No*/*Yes*). The robot use self-efficacy scale was based on the RUSH-3 scale (Turja et al., 2019) and included three statements on a scale from 1 (*strongly disagree*) to 7 (*strongly agree*). The statements used in this study were (a) “I’m confident in my ability to learn how to use robots,” (b) “I’m confident in my ability to learn the simple programming of robots if I were provided the necessary training,” and (c) “I’m confident in my ability to learn how to use robots in order to guide others to do the same.” The measure showed good reliability based on Cronbach’s alpha (α = 0.88), and the final sum variable ranged from 3 to 21. Previous experience with robot use was determined with a single-item question: “Have you ever used a robot or interacted with a robot?”

#### Personality

For the second part of the study, we measured personality traits with a 15-item Big Five inventory, in which participants scored statements on a scale from 1 to 7 (Lang et al., 2011). For each personality trait, we created a 3-item sum variable ranging from 3 to 21. Interitem reliability figures ranged from *good* to *acceptable*: neuroticism (α = 0.85), extroversion (α = 0.84), openness (α = 0.79), agreeableness (α = 0.62), and conscientiousness (α = 0.67).

#### Statistical Techniques

We conducted all analyses with Stata 16. Because violation of normality was moderate and our sample was large (*N* = 1077), we decided to run analyses using a parametric one-way and two-way ANOVA. Negative kurtosis is not considered a problem with larger samples (Waternaux, 1976; see also Tabachnick and Fidell, 2013; Gravetter and Wallnau, 2017). In addition, our experiment and control groups were relatively equal in size. Also, the results of Bartlett’s test for equal variance were insignificant, indicating that experiment and control groups had similar variation (χ^2^[5] = 2.75, *p* = 0.739). We conducted a robustness check by running a non-parametric Kruskal–Wallis *H* test. As these results showed no deviation from the parametric tests, we only report the parametric one-way and two-way ANOVA tests.

The second part of the study was based on ordinary least squares regression. Unstandardized regression coefficients (*B*) and their standard errors (*B SE*), standardized beta coefficients (β), *p-*values, model goodness-of-fit measures (*R*^2^), model test (*F*), and *p-*values were reported. We did not detect problematic multicollinearity. A Breusch–Pagan test for heteroskedasticity showed no problems with heteroskedasticity of residuals (χ^2^ = 0.65, *p* = 0.42). Residuals were also considered as having normal distribution (skewness = 0.22, kurtosis = 0.237 when 3 = normal distribution). We detected outliers by looking at Cook’s distance measure, where values greater than 4/N may cause problems. Due to the existing outliers, we also ran the model with a robust regression considered to be a solution for cases in which outliers are present (Verardi and Croux, 2009). The results by robust regression run with a rreg command in Stata are reported in the Appendix B, as they did not change any of the results.

## Results

**FIGURE 1:**
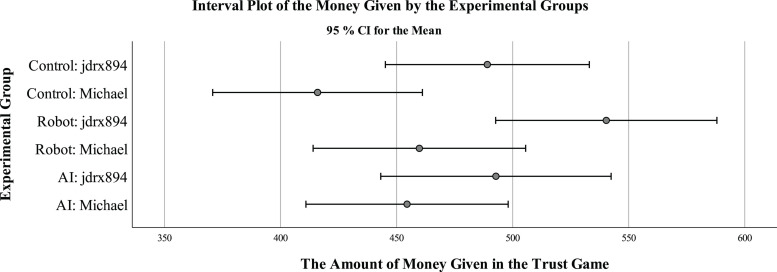
Trust game mean values (95% CI) on a scale of 0–1000 by experimental groups in Study 1 (*N* = 1077).

The descriptive statistics in Table 1 and Figure 1 show that the opponent described as “jdrx894, a robot” was given the highest sum of money on average, and the lowest sum of money was given to the opponent called Michael. The one-way ANOVA results for the six groups showed statistically significant differences between groups [*F*(5,1071) = 3.17, *p* = 0.008]. A pairwise comparison of means using Tukey’s honest significant difference test indicated that jdrx894 robot (*M* = 540.33), received more money than Michael (*M* = 415.95, *p* = 0.003) in the control group.

**TABLE 1:** Descriptive statistics on the amount of money given in the experimental and control groups in Study 1 (*N* = 1077).

Group	*n*	%	*M*	*SD*	*Md*	Range
AI: Michael	185	17.18	454.50	300.44	500	0–1000
AI: jdrx894	171	15.88	492.78	328.83	500	0–1000
Robot: Michael	192	17.83	459.80	321.58	478	0–1000
Robot: jdrx894	172	15.97	540.33	316.55	500	0–1000
Control: Michael	171	15.88	415.95	299.23	450	0–1000
Control: jdrx894	186	17.27	489.08	303.74	500	0–1000

Two-way ANOVA was run to analyze the effect of name (Michael or jdrx894) and type of the opponent (robot, AI or control) (see Table 2). There were no statistically significant differences between three types of opponents. Opponents called Michael were trusted less, *F*(1,1071) = 11.31, *p* = 0.001. Analysis of adjusted means based on the ANOVA model showed that jdrx894 received $507.40, but Michael only received $444.42.

**TABLE 2:** Two-way analysis of variance of money given in the trust game in the experimental groups and control groups in Study 1 (*N* = 1077).

Measure	df	MS	*F*	*p*	$\eta_{\text{p}}^{2}$
Type	2	204267.63	2.10	0.123	–
Name	1	1099834.3	11.31	0.001	0.01
Type × name	2	45485.881	0.47	0.627	–
Residual	1,071	97262.353			
Total	1,076	98244.876			

*Name (Michael or jdrx894) and type (robot, AI or unspecified control) refer to the opponents; MS, mean squares.*

The second part of the analysis was focused on analyzing the correlations of the trust expressed in robots and AI (*n* = 720). Participants in robot and AI conditions were combined due to the fact that previous analysis showed no statistically significant differences between them. Results were also similar in regression models and there were no statistically significant interactions between conditions. The average sum of money given to the robot or AI opponents was $485 (*M* = 485.51, *SD* = 318.00, range $0–$1,000). Table 3 contains details on independent variables, excluding control groups that were not used in the second part of the analysis. There were some notable differences between participants. For example, those with degrees in engineering or technology gave an average of $530, and others gave an average of $471. The regression model shown in Table 4 further analyzes which sociodemographic and social–psychological factors were associated with giving money to an opponent. The model was statistically significant, and the included variables explained 9% of the variance (*R*^2^ = 0.09, *F* = 4.90, *p* < 0.001).

**TABLE 3:** Descriptive statistics of independent variables used for regression analyses (*N* = 720).

*Categorical measures*	*n*	%			
**Age**					
<40	484	67.22			
40 and over	236	32.78			
**Gender**					
Female	353	49.03			
Male	357	49.58			
Other/not specified	10	1.39			
**Occupational status**					
Student	20	2.78			
Works full or part time	611	84.86			
Other	89	12.36			
**Household’s gross annual income**					
<$35,000	190	26.39			
$35,000–$154,999	495	68.75			
$155,000 and over	35	4.86			
**Technology/engineering degree**					
No	536	74.44			
Yes	184	25.56			
**Robot exposure online**					
No	345	47.92			
Yes	375	52.08			

***Continuous measures***	***M***	***SD***	**Range**	***n* of items**	**α**

**Robot use self-efficacy**	16.09	3.64	3–21	3.00	0.88
**Personality traits**					
Neuroticism [Big Five]	10.76	5.14	3–21	3.00	0.85
Extraversion [Big Five]	11.32	4.81	3–21	3.00	0.84
Openness [Big Five]	15.36	3.81	3–21	3.00	0.79
Agreeableness [Big Five]	15.35	3.67	3–21	3.00	0.62
Conscientiousness [Big Five]	16.22	3.39	3–21	3.00	0.67

**TABLE 4:** Linear regression analysis on money given to an AI or a robot opponent (*N* = 710).

Measure	*B*	*SE B*	*p*	β
Age over 40	91.33	25.64	<0.001	0.13
Female	−27.55	24.74	0.266	−0.04
Occupational status				
Student	Ref.	Ref.	Ref.	Ref.
Works full or part time	−11.65	70.64	0.869	−0.01
Other	−48.71	77.14	0.528	−0.05
Household’s gross annual income				
<$35,000	−58.16	27.32	0.034	−0.08
$35,000–$154,999	Ref.	Ref.	Ref.	Ref.
$155,000 and over	−95.11	54.06	0.079	−0.06
Technology/engineering degree	58.85	29.37	0.045	0.08
Robot exposure online	47.65	23.86	0.046	0.07
Robot use self-efficacy	14.22	3.58	<0.001	0.16
Neuroticism [Big Five]	1.73	2.66	0.516	0.03
Extraversion [Big Five]	0.92	2.63	0.727	0.01
Openness [Big Five]	7.80	3.41	0.022	0.09
Agreeableness [Big Five]	1.08	3.66	0.768	0.01
Conscientiousness [Big Five]	−12.79	4.23	0.003	−0.14

*Those not identified as males or females (*n* = 10) were dropped from the model for estimation reasons.*

Age (40 years or more; β = 0.13, *p* < 0.001) and technology/engineering degree (β = 0.08, *p* = 0.045) were also associated with giving money to robots and AI when adjusting for a number of other factors. We also noted that participants in both low- and high-income brackets gave less money to robot and AI opponents. However, we found statistical significance only when comparing the $35,000–$154,999 income group to households with a gross annual income of less than $35,000 (β = −0.08, *p* = 0.034).

The findings also indicated that participants who were exposed to robots online gave more money to robots and AI opponents (β = 0.07, *p* = 0.046). The single most important predictor for giving money to an AI or robot was robot use self-efficacy (β = 0.16, *p* < 0.001). Personality traits of neuroticism, extroversion, and agreeableness were not statistically significant. However, those showing openness to experiences gave more money to robot or AI opponents (β = 0.09, *p* = 0.022), and those showing conscientiousness gave less money (β = −0.14, *p* = 0.003).

We used the last model to seek the potential exposure effects of previous experience with robots. One-third of participants (33.19%) reported such experience. The interaction term between robot use experience and robot use self-efficacy was added to the model. The model was statistically significant, and 10% of the variance was explained (*R*^2^ = 0.10, *F* = 4.60, *p* < 0.001). Results showed that the variables that were statistically significant in the previous model remained so (see Appendix C). Thus, noting previous robot use experience did not change the results in any way. However, the interaction term was negative (β = −0.40, *p* = 0.038), indicating that those who had robot use experience and high robot use self-efficacy gave lower sums of money to AI and robot opponents than those without previous experience with robots.

## Discussion

Trust is a crucial dimension in human–technology interaction. We investigated the extent to which participants trust robots or AI using a trust game experiment. We found out that, contrary to our hypotheses, opponent type (robot, AI or not specified control) had no significant effect on trust. However, opponents named jdrx894 were trusted more than those named Michael. The most trusted opponent was the robot jdrx894, and least trusted was Michael in the control group.

Hypotheses of the second part of our study were mostly confirmed, as we expected that technology education, online robot exposure, and robot use self-efficacy would predict trust toward robots and AI. The results were in line with previous research about the relevance of user experience and familiarity with robotics (Hancock et al., 2011) and robot use self-efficacy (Latikka et al., 2019). Exposure to online discussions might also have a positive impact on trust. Yet, this matter needs to be investigated in other studies, as research on this topic is scarce. However, the potential impact of online communities and discussions has been noted before (Moorhead et al., 2013; Keipi et al., 2017).

Our results also underline the relevance of personality in understanding human–technology interactions and trust. Based on the hypotheses concerning personality traits, we found evidence for a positive correlation between openness and trust toward robots and AI, which is in line with previous research related to personality factors and trust in general (Kaplan et al., 2015). In contrast to what Chien et al. (2016) found in their study about trust toward automation, our results suggest a negative association between conscientiousness and trust toward robots and AI. We found no relationship concerning the traits of agreeableness, extraversion, or neuroticism.

The results make sense from the perspective that people have become more exposed and accustomed to robots over time. Also, robots have been designed to be more attractive, approachable, and predictable based on, for example, gestures (e.g., Li et al., 2010; Sanders et al., 2011; Mou et al., 2020), whereas AI’s image may be more abstract and distanced, although similar design attempts have been made in combining AI bots with humanlike virtual images (Araujo, 2018). Rich visual mental imagery has been found to affect how robots are perceived (Fortunati et al., 2015), and for these reasons, it might be easy for participants to trust a robot called jdrx894. According to the results of the experiment, participants showed lowest trust to the control group Michael who could be interpreted as another human. This could be explained by studies showing that people tend to consider others more selfish and negative than they actually are (Vuolevi and Van Lange, 2010; Van Lange, 2015). For these reasons, our participants might have been willing to think that robots are trustworthy. This would contrast the similarity–attraction hypothesis (Nass and Lee, 2001; Montoya and Horton, 2013). Another issue is gender. Our experiment included only a male opponent named Michael. This might have impacted the results, as males are generally perceived as less trustworthy than females in economic game experiments (Bohnet and Zeckhauser, 2004; Buchan et al., 2008).

Visual anonymity was an aspect of the experiment. We did not include any pictures or information about AI or robots because we wanted to measure the minimal conditions that might impact behavior in interactional settings, such as online customer service encounters. Our results based on a sample of participants from the United States suggest that software developers and service providers should not hide the true identity of intelligent non-human agents. Visual anonymity in the experimental context might also have an impact on behavior. In this type of experiment, players might consider the situation such that they would not ever meet the opponent again. This is different from normal face-to-face encounters in everyday life, where trust or distrust of others might carry long-term consequences.

The analysis showed additional evidence for trust toward robots and AI. In line with established theories and empirical evidence (Hancock et al., 2011; Van Lange, 2015; Schaefer et al., 2016), predictors of trust were having a degree in technology or engineering, having prior experience and self-efficacy with robots, and exhibiting openness as a personality trait, confirming the hypotheses, for the most part. We also noted that exposure to online robot discussions predicted trust. It was interesting, however, that although prior experience has been found to associate with the acceptance of robots (Venkatesh, 2000; Nomura et al., 2006; Bartneck et al., 2007; Heerink et al., 2010), we found an interaction effect indicating that those who had robot use experience and high robot use self-efficacy gave lower sums of money to AI and robot opponents than those who did not have experience with robots. This interaction also reveals that despite being familiar with robotics, people might be also skeptical of intentions of robots and AI with higher skills. More studies on trust are needed from this perspective.

Our study is based on a minimal condition, giving few cues about the nature of robots and AI. Such minimal conditions are important, especially when analyzing trust and behavior online, where various cues are left out. Our control groups used only the human name Michael and nickname jdrx894 without describing them explicitly as humans, because we did not want to indicate to the participants that opponents might not be humans. This resulted in a reliable control condition. However, this decision is also a limitation of the study, as we cannot be sure that all participants interpreted the control group opponents as humans. Further limitations of our study were that there was no manipulation check to ensure that participants had paid attention to names or descriptions of opponents as robots or AI, or a check to ensure whether participants had understood how to maximize their gains on the trust game.

Future studies could, however, describe one of the experimental groups explicitly as humans. It would also be good to use female names, as the male name used in our study was considered less trustworthy than the nickname. It might also be possible to conduct an experiment with various types of robots and AI avatars using trust game settings. In addition, more studies on individual factors, such as personality, would be needed, as our results showed that they impacted trust. These types of factors could be crucial when introducing new technologies to people.

Robots and AI were not less trusted than the control group, which indicates that people are becoming more trusting toward new technology, at least in contexts where one needs to be able to trust the cognitive abilities and fairness of advanced technology. In other words, our results suggest that in some conditions, technological entities can be perceived as rational actors that, without hidden motivations and agendas, make more sensible and unselfish decisions than humans. This has potentially major implications for a variety of service sector fields. This finding can also be understood from a broader perspective, as a shift toward a “feeling economy” as the next generation of AI (Huang et al., 2019). People are currently impacted by public discussions about robots, and this was evident in our results, indicating that those who were more familiar with online robots showed more trust. However, we determined that those with prior experience using robots and very high robot use self-efficacy were not necessarily trusting. This hints that the current development of AI may also cause concern, for example, about the capabilities of machine learning and its ethical regulation among the most technologically knowledgeable and capable individuals.

## Data Availability Statement

The raw data supporting the conclusions of this article will be made available by the authors, without undue reservation.

## Ethics Statement

The studies involving human participants were reviewed and approved by The Ethics Committee of the Tampere Region. The participants provided their informed consent to participate in this study.

## Author Contributions

AO contributed to the conceptualization, data collection, investigation, methodology, formal analysis, writing original draft, supervision, and funding acquisition. NS contributed to the conceptualization, data collection, investigation, methodology, reviewing and editing the manuscript, and visualization. RL contributed to the conceptualization, investigation, reviewing and editing the manuscript. AK contributed to the methodology, investigation, and review and editing the manuscript. All authors contributed to the article and approved the submitted version.

## Conflict of Interest

The authors declare that the research was conducted in the absence of any commercial or financial relationships that could be construed as a potential conflict of interest.

## Appendix A

The following is an example of information given for the trust game study participants.

We now invite you to participate in an imaginary game. In the game you play against another player, and you will make decisions about how to distribute a sum of money between the two of you.

*At the beginning of the game, you receive $1,000. You can decide whether you keep this whole sum to yourself or whether you share a part or all of it with the player that you are playing against. If you give money to the other player, we will triple the sum that you give. So, if you give $500, the other player receives $1,500. Then, it is up to the other player to decide how much money to return to you. As an example, if the person returns half, you will end up with $500* + *$750* = *$1,250 at the end of the game. The other player may also choose not to return anything, which would mean that you win only the $500 that you kept to yourself from the beginning.*

The more money you obtain, the more successful you will be!

Name of the opponent: Michael, an artificial intelligence.

Fill in the amount in this box (between $0–$1,000).

## Appendix B

**TABLE A1:** Robust regression analysis on money given to an AI or a robot opponent (*N* = 710).

Measure	*B*	*SE B*	*p*	95%	CI
Age over 40	104.20	27.53	<0.001	50.14	158.25
Female	−31.26	26.57	0.240	−83.43	20.90
Occupational status					
Student	Ref.	Ref.	Ref.	Ref.	Ref.
Works full or part time	−20.18	75.87	0.790	−169.14	128.78
Other	−60.77	82.85	0.463	−223.44	101.89
Household’s gross annual income					
<$35,000	−62.68	29.34	0.033	−120.29	−5.07
$35,000–$154,999	Ref.	Ref.	Ref.	Ref.	Ref.
$155,000 and over	−105.69	58.06	0.069	−219.68	8.29
Technology/engineering degree	69.82	31.54	0.027	7.89	131.75
Robot exposure online	47.94	25.62	0.062	−2.37	98.25
Robot use self-efficacy	15.06	3.84	<0.001	7.52	22.60
Neuroticism [Big Five]	1.97	2.86	0.491	−3.65	7.59
Extraversion [Big Five]	1.20	2.82	0.671	−4.35	6.75
Openness [Big Five]	8.70	3.66	0.018	1.52	15.88
Agreeableness [Big Five]	1.58	3.93	0.687	−6.12	9.29
Conscientiousness [Big Five]	−13.74	4.54	0.003	−22.66	−4.82

*Those not identified as males or females (*n* = 10) were dropped from the model for estimation reasons.*

## Appendix C

**TABLE A2:** Additional linear regression analysis on money given to an AI or a robot opponent (*N* = 710).

Measure	*B*	*SE B*	*p*	β
Age over 40	88.23	25.63	0.001	0.13
Female	−29.98	24.74	0.226	−0.05
Occupational status				
Student	Ref.	Ref.	Ref.	Ref.
Works full or part time	−4.18	70.74	0.953	0.00
Other	−38.63	77.38	0.618	−0.04
Household’s gross annual income				
<$35,000	−55.18	27.39	0.044	−0.08
$35,000–$154,999	Ref.	Ref.	Ref.	Ref.
$155,000 and over	−95.22	54.06	0.079	−0.06
Technology/engineering degree	65.62	29.92	0.029	0.09
Robot exposure online	47.72	23.89	0.046	0.07
Robot use experience	247.51	129.09	0.056	0.37
Robot use self-efficacy	18.16	4.01	<0.001	0.20
Neuroticism [Big Five]	1.73	2.66	0.516	0.03
Extraversion [Big Five]	1.05	2.63	0.690	0.02
Openness [Big Five]	7.87	3.40	0.021	0.09
Agreeableness [Big Five]	1.17	3.65	0.749	0.01
Conscientiousness [Big Five]	−12.50	4.24	0.003	−0.13
Robot use experience × robot use self-efficacy	−15.88	7.63	0.038	−0.40

*Those not identified as males or females (*n* = 10) were dropped from the model for estimation reasons.*
